# Antioxidant Potential of Fruit Juice with Added Chokeberry Powder (*Aronia melanocarpa*)

**DOI:** 10.3390/molecules22122158

**Published:** 2017-12-05

**Authors:** Jana Šic Žlabur, Nadica Dobričević, Stjepan Pliestić, Ante Galić, Daniela Patricia Bilić, Sandra Voća

**Affiliations:** Department of Agricultural Technology, Storage and Transport, Faculty of Agriculture, University of Zagreb, Svetošimunska Cesta 25, 10 000 Zagreb, Croatia; jszlabur@agr.hr (J.Š.Ž.); ndobricevic@agr.hr (N.D.); spliestic@agr.hr (S.P.); dpbilic@gmail.com (D.P.B.); svoca@agr.hr (S.V.)

**Keywords:** anthocyanins, polyphenolic compounds, classic extraction, ultrasound assisted extraction, antioxidant capacity

## Abstract

The purpose of this study was to determine the possibility of using chokeberry powder as a supplement in apple juice to increase the nutritional value of the final product with the aim of developing a new functional food product. Also, to determine the influence of ultrasound assisted extraction on the bioactive compounds content, nutritional composition and antioxidant potential of apple juice with added chokeberry powder. The juice samples with added chokeberry powder had higher antioxidant capacity, irrespective of the extraction technique used. Apple juice samples with added chokeberry powder treated with high intensity ultrasound had significantly higher content of all analyzed bioactive compounds. The application of high intensity ultrasound significantly reduced the extraction time of the plant material. A positive correlation between vitamin C content, total phenols, flavonoids and anthocyanins content and antioxidant capacity was determined in juice samples with added chokeberry powder treated with high intensity ultrasound.

## 1. Introduction

Fresh berries, including various chokeberry products, have recently gained great popularity among consumers primarily because their high health value. According to the ORAC scale, chokeberry possesses the highest antioxidant activity value among other berry and fruit species [1,2,3]. By nutritional composition, fresh chokeberry is a rich source of bioactive compounds such as vitamin C, polyphenolic compounds, flavonoids and anthocyanins. The dark red coloration of chokeberry fruit is a result of the presence of anthocyanin, because of which chokeberry can be used as a natural dye. The most common chokeberry products are powder, syrup, juice, fruit jelly, fruit tea, liquor and wine. Consumption of juices and other chokeberry products is often limited due to its unique bitter, astringent taste so they are rarely used in their original form, but are more often added to other fruit products [4]. One of the possibility of chokeberry powder application is addition to various juices of other fruit species frequently to increase the organoleptic (specifically product color) and nutritional characteristics of the final product. In daily consumption one of the most popular fruit juices is apple juice while in food industry apple juice is often used as a base element in the preparation of other fruit products mainly because it’s rich content of phytochemicals with strong antioxidant activity [5]. Based on the significant bioactive compounds content and high antioxidant activity, apple juice enriched with chokeberry powder potentially presents a new product type that may be categorized as a functional food. 

Nowadays, in the field of food technology and biotechnology, new processes are evolving with the main aim of preserving organoleptic and nutritional qualities of the final product. Also, consumer demands are increasingly focused on high-quality, health and hygienically proper products, so the above-mentioned technology processes are focused to the principles of green chemistry, respectively environmentally and health-friendly chemical processes [6]. High intensity ultrasound recently found a great potential in processing technologies such as: drying, filtration, extraction, inactivation of microorganisms, homogenization, etc. [7]. High intensity ultrasound is characterized by the use of high intensity acoustic waves (typically in the range from 10 to 1000 W/cm^2^) and the frequencies between 18 and 100 kHz [7,8] during which in the liquid medium occurs phenomenon of transient cavitation. Ultrasound assisted extraction (UAE) is one of the most applicable and numerous researches cites it’s efficiency and other positive benefits such as reduced time of extraction, increased yield of different chemical compounds (polyphenols, vitamins) and significant energy savings [9,10,11].

The aim of this study was to determine the possibility of using the chokeberry powder as a supplement in apple juice to increase the nutritional value of the final product with a potential view to developing a new functional food product. Also, to determine the influence of ultrasound assisted extraction on the bioactive compounds content, nutritional composition and antioxidant potential of apple juice with added chokeberry powder.

## 2. Results

### 2.1. Basic Chemical Composition

The basic chemical composition results of raw apple juice (sample A), fruit juice samples with added chokeberry powder extracted classically (B1 to B7) and by those exposed to high intensity ultrasound (C1 to C6) are shown in Table 1.

**Figure 1:**
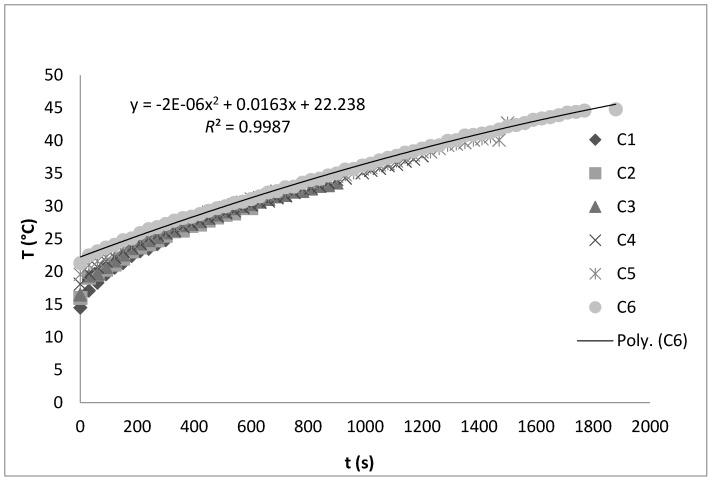
Temperature change in juice samples during ultrasonic treatment.

ANOVA assay showed high significant statistical differences (*p* ≤ 0.0001) between all analyzed samples (A, B, C) depending on the extraction method. The solution density of the analyzed juice samples with added classically extracted chokeberry powder amounted on average to 1.0538 g cm^−3^, while for juice samples extracted by ultrasound the average density was 1.0543 g cm^−3^. One of the major consequences of high intensity ultrasound is a transient cavitation phenomenon which increases the temperature in the system affecting a range of physical properties of the treated solutions, including density [8]. In juice samples with added chokeberry powder treated by ultrasound for longer time periods (15–30 min) the highest density values were determined, which was expected considering the recorded temperature increase of the system (Figure 1).

The average amount of total soluble solids (TSS) in juice samples with added chokeberry powder extracted classically was 13.99%, while in juice samples treated by ultrasound the average content was 14.05%. In general, the total soluble solids content in juice samples with added chokeberry powder shows an increasing trend in all the varied time periods, regardless of the extraction method. Extraction method, including high intensity ultrasound treatment, did not significantly affect the TSS in analyzed juice samples. In juice samples with added chokeberry powder extracted classically the average total acid (TA) content was 17.44%, while in samples treated by ultrasound the average TA was 17.68%, which for both extraction methods was approximately 2% higher than the control sample (A) value. TA content does not show significant differences depending on the extraction method (classic and ultrasound). It is important to emphasize that the addition of chokeberry powder in apple juice significantly increased the TA content regardless of the extraction method which was expected given the high vitamin C content determined in juice samples with added chokeberry powder. According to the Tolić et al. [4] TA content in chokeberry ranges from 0.29% to 1.32%, Kulling and Rawel [1] cite values in a range from 0.86% to 0.99%, while Jeppsson [12] and Šnebergová et al. [13] cite TA values in fresh berries in the range from 0.67% to 1.19%. pH-values of analyzed juice samples treated classically or by ultrasound of high intensity did not differ significantly, i.e., the pH-values of control sample as well as samples with added chokeberry powder extracted classically (B1–B7) and by ultrasound (C1–C6) are not significantly different (*p* ≤ 0.5321). The average pH-value of all classically treated samples is 3.35 while for samples treated by ultrasound it is 3.36, which in comparison with the control sample (A) is a negligible difference.

### 2.2. Bioactive Compounds Content

The bioactive compounds content results of juice samples with added chokeberry powder are shown in Table 2. High significant statistical differences (*p* ≤ 0.0001) were determined between all analyzed juice samples (A, B, C) depending on the method and time period of extraction. Fresh chokeberry fruit, as well as different chokeberry products such as juice and powder are rich sources of vitamin C [1,14,15], which has also been confirmed in this study. Namely, the determined vitamin C content in control sample (A) was 13.16 mg 100 g^−1^ while in juice samples with added chokeberry powder it was significantly higher, regardless of extraction method, with the exception of sample B1 which didn’t significantly differ in vitamin C content from control sample (Table 2). The duration of the extraction period in both extraction methods (classic and ultrasound) significantly affected the vitamin C yield increase; thus classic extraction from 5 min to 24 h and ultrasound extraction from 5 to 30 min increased the vitamin C content about 3-fold. The highest vitamin C content was determined in the juice sample treated by ultrasound of high intensity for 30 min (C6) which is a 49% higher value compared with the same time period of classic extraction (sample B6), and a 16% higher value compared with sample B7 (24 h of classic extraction). 

Temperature increase during ultrasonic treatment enhances the diffusion process [16] while at the same time does not cause vitamin C degradation in the juice samples. Chokeberry is also characterized by high content of polyphenols, anthocyanins and flavonoids [4]. The total phenols content between all the studied juice samples (A, B, C) was significantly statistically different (*p* ≤ 0.0001). Addition of chokeberry powder significantly increased the total phenol content regardless of the extraction method (classical and ultrasound, Table 2). Total phenol content in juice samples in which chokeberry powder after classical and ultrasound extraction shows an increasing trend for all the varied time periods. The most significant differences in the content of total phenols were determined in juice samples treated by ultrasound for 30 min: compared to the control sample (A) the total phenol increase was as high as 86%, in comparison with the juice sample B6 (classic extraction for 30 min) which increase was 12% and 25% in comparison with the juice sample B7 in which the powder was extracted classically for 24 h. The positive effect of high intensity ultrasound on the phenolic compounds extraction is in accordance with other literature data which emphasizes the significant efficacy of high intensity ultrasound on the extraction of different chemical compounds with various molecule structures [10,11,17,18]. In chokeberry fruits flavonoids (quercetin glycosides) are the most common from the group of polyphenols [2,14,15,19,20,21,22,23]. In all analyzed juice samples high statistical differences (*p* ≤ 0.0001) of flavonoids content were determined (Table 2). Significant difference was determined between the juice sample with added chokeberry powder extracted by ultrasound for 30 min (C6) and the juice sample extracted classically for 24 h (B7). The determined difference between samples C6 and B7 amounted to 15%. Also, other scientific studies have proven the significant efficiency of high intensity ultrasound in the extraction of flavonoids [9,10,11]. Dark red chokeberry coloration is the result of the presence of anthocyanin which is primarily known for its strong antioxidant activity. According to the results from Table 2 juice samples with added chokeberry powder extracted with ultrasound for 30 min (C6) had 84% higher anthocyanin content compared to the juice sample extracted classically for the same time period (B6). Anthocyanin yield increased significantly depending on the extraction time, from 5 min to 24 h. Also, an increasing trend of anthocyanin content was recorded in the juice samples treated by high intensity ultrasound.

Other authors have presented results contrary to those shown in this study, emphasizing the degradation effect of high intensity ultrasound on the total anthocyanins content [24,25,26,27]. The main factors of high intensity ultrasound causing degradation of anthocyanins content are the amplitude and the extraction time. At higher levels of ultrasonic amplitude and prolonged time periods a higher degradation level of anthocyanins content was determined [28,29,30]. Also, another important factor of the ultrasound effect is the temperature increase; high temperatures have a significant impact on the anthocyanin reduction [27,28]. In this study, relatively low ultrasound power levels as well as a not-so-significant temperature increase in the system (max 44.8 °C, Figure 1) did not cause a significant reduction of the analyzed bioactive compounds content. Besides total bioactive compounds content, the content of the most common individual anthocyanins and other compounds from the group of polyphenols were analyzed (Table 3). High significant statistical differences (*p* ≤ 0.0001) for all analyzed individual polyphenolic compounds depending on the method and extraction time were determined. The addition of chokeberry powder to apple juice, besides anthocyanins, significantly increased the content of other studied polyphenols, specifically the content of chlorogenic acid and quercetin. Extraction time from 5 min to 24 h positively influenced the yield increase of all analyzed compounds in both applied extraction methods, classic and ultrasound. High intensity ultrasound showed a positive effect on the yield of the analyzed individual anthocyanins: cy 3-galactoside, cy 3-glucoside, cy 3-arabinoside i cy 3-xyloside. Namely, comparing the juice sample with added chokeberry powder extracted classically for a time period of 30 min (B6) and the juice sample extracted for the same time period by high intensity ultrasound (C6) increases of cy 3-galactoside and cy 3-arabinoside by 2 times, cy 3-glucoside by 46%, and cy 3 xyloside by 86% were determined. Also, it is important to emphasize that a higher yield of all analyzed individual anthocyanins was identified in the sample C6 compared to the sample B7in which the extraction of mentioned anthocyanins lasted significantly longer (24 h) in the order of: cy 3-galactoside 19%, cy 3-glucoside 11%, cy 3-arabinoside 24% and cy 3-xyloside 18%. The positive impact of high intensity ultrasound and time period of extraction is also determined for other studied individual polyphenolic compounds. Chlorogenic acid content in sample C6 was 90% higher compared to sample B6 (classically extracted for 30 min), the epicatechin content increased by 98%, quercetin by 94% while the myricetin content did not change significantly.

### 2.3. Antioxidant Capacity

Among all studied juice samples significant statistical differences (*p* ≤ 0.0001) for antioxidant capacity were determined (Table 2). The lowest antioxidant capacity was determined in control sample (A) which is expected given that mentioned sample does not contain chokeberry powder. Time period as well as extraction technique showed a positive impact on the increase of antioxidant capacity in all juice samples, except for sample B7 in which a lower antioxidant capacity in comparison with samples B1 to B6 was determined. The mentioned results suggest that too long an extraction period can cause an opposite effect on bioactive compounds content, and particularly antioxidant capacity. The antioxidant activity of plant species is directly correlated with the content of vitamins, pigments and various plant phenolic phytochemicals, such as flavonoids, glycosides, alkaloids and others [10,31]. By statistical analysis of the correlations, a relationship between the two variables was observed: between the bioactive compounds content (vitamin C, total phenols, total flavonoids and total anthocyanins) and antioxidant capacity in the juice samples treated classically (B1 to B7) and juice samples treated by high intensity ultrasound (C1 to C6) (Table 4). In the classic treatments of the chokeberry powder the significance of the coefficient was determined between the content of total phenols and antioxidant capacity, while for the other observed parameters the significance was not determined. The significance of correlation coefficient was determined between all chemical parameters and antioxidant capacity in juice samples with added chokeberry powder treated with high intensity ultrasound for which correlation coefficient (*r*) were: *r* = 0.79 (total flavonoids), *r* = 0.82 (total phenols), *r* = 0.83 (vitamin C), *r* = 0.86 (total anthocyanins). Obtained results suggest that juice samples with higher determined vitamin C, total phenols, flavonoids and total anthocyanins content showed higher antioxidant capacity. Juice samples treated with high intensity ultrasound for 30 min had the highest content of all studied biologically active compounds and thus the highest antioxidant capacity. Based on the results of correlation coefficients it can be concluded that a stronger positive correlation exists between the analyzed bioactive compounds and antioxidant capacity in juices samples treated by ultrasound compared with the juice samples in which the chokeberry powder was extracted classically.

## 3. Materials and Methods

### 3.1. Plant Material

Chokeberry fruits (*Aronia melanocarpa*) were obtained from the Department of Pomology, Croatian Centre for Agriculture, Food and Rural Affairs (Zagreb, Croatia). Berry harvest was carried out at optimum fruit maturity, which occurs around the end of August. After the harvest the fruits were transported to the laboratory of the Department of Agricultural Technology, Storage and Transport, Faculty of Agriculture, University of Zagreb where they were washed and fruits with any mechanical damages and spoilage were discarded. 

### 3.2. Chokeberry Powder Preparation

Fruit juice was isolated from the chokeberry fruits by a thermal heating process. The remaining fruit pulp was dried by a process of convective drying in a laboratory dryer (INKO ST 40, Zagreb, Croatia) at 60 °C until a water content of 10% was achieved. Dried chokeberry pulp was milled to a powder by a laboratory mill (IKA MF-10, Staufen, Germany) and stored in dark glass packaging in a dark, dry place. Total dry matter content of dried chokeberry powder was determined by a standard method of drying at 105 °C [30] and amounted to 9.65%. Apple juice was purchased in a local market for research purposes. The apple juice was RICO brand (Darda, Croatia) produced from ‘Golden Delicious’ cultivar apples by a pressing process.

### 3.3. Sample Preparation for the Classic Extraction

The experimental design of classical extraction is shown in Table 5. A previously weighed amount of chokeberry powder (2.5 g ± 0.0001 g) was placed in a 250 mL laboratory flask. On this chokeberry powder, room temperature (21.4 °C) apple juice (100 mL) was added. These samples were allowed to stand at room temperature for: 5 min (sample B1), 10 min (sample B2), 15 min (sample B3), 20 min (sample B4), 25 min (sample B5), 30 min (sample B6) and 24 h (sample B7). After each time period, the samples were filtered through Whatman filter paper (pore size 8–12 µm) to remove chokeberry powder and to stop further extraction. 

### 3.4. Sample Preparation for Ultrasonic Extraction

The experimental design for ultrasonic assisted extraction is shown in Table 5. Prior to ultrasonic treatment, chokeberry powder (2.5 g ± 0.0001 g) was weighed into a glass beaker (250 mL) and room temperature (21.4 °C) apple juice (100 mL) was added. Immediately after the juice addition ultrasonic treatment was carried out, during which the time periods were varied as follows: 5 min (sample C1), 10 min (sample C2), 15 min (sample C3), 20 min (sample C4), 25 min (sample C5) and 30 min (sample C6). After each time period samples were filtered. Ultrasonic extraction was carried out in an ultrasonic bath (Bandelin RK 103H, Berlin, Germany) at a frequency of 35 kHz and a nominal maximum power of 140 W. Also, during each ultrasonic treatment the temperature of the samples was measured at 30 s time intervals with an infrared thermometer (Uni-Trend Technology UT 300C, Dongguan, China). This data is shown in Figure 1. 

### 3.5. The Determination of Basic Chemical Composition and Bioactive Compounds Content

The following chemical analysis for determination of basic chemical composition were carried out: solution density (g cm^−3^) by a digital densitometer (Mettler–Toledo Densito 30PX, Schwerzenbach, Switzerland), total soluble solids content (%) by a digital refractometer (Mettler–Toledo Refracto 30PX) [30], total acid content (%) by potentiometric titration [30], pH–value by a digital pH–meter (Mettler–Toledo SevenMulti, Schwerzenbach, Switzerland) [30]. For determination of total content of specific bioactive compounds following analysis were carried out: total phenols and flavonoids content (mg L^−1^) were obtained according to [31], total anthocyanins (mg L^−1^) by bisulfite bleaching [32]. The antioxidant capacity was determined by ABTS method [33].

### 3.6. Vitamin C Determination (HPLC Method)

The analytical HPLC system employed consisted of a 920 LC system (Varian, Melbourne, Middelburg, Australia) equipped with Galaxie software (Varian, Melbourne, Australia), a multiple UV wavelength detector, auto-injector, autosampler and quaternary pump. Separation was done on Nucleosil C-18, 5 µm (250 × 4.6 mm I.D.) column with a Nucleosil C-18 guard column, 5 µm (10 × 4.6 mm I.D.). Juice samples were filtered through Nylon filter (0.45 µm) and directly injected into vials. The HPLC method for identification of vitamin C content was performed according to the Odriozola-Serrano et al. [34]. The employed mobile phase was sulfuric acid solution 0.01% (pH 2.6) at a flow rate of 1 mL min^−1^ with isocratic elution. Operating conditions were: column temperature 20 °C, injection volume 10 µL of the standards and extract samples. The detector was set at 245 nm. For identification a standard of vitamin C (Sigma Aldrich, St. Louis, MO, USA; Steinheim, Germany) was used. 

### 3.7. HPLC Determination of Individual Anthocyanins and Polyphenols

For the HPLC determination of individual anthocyanins and polyphenols the same Varian 920 LC HPLC equipment as for vitamin C determination was used. Juice samples were filtered through a nylon filter (0.45 µm) and directly injected into vials. The HPLC methods for identification of individual anthocyanins and polyphenols were performed according to the Jakobek et al. [35]. For anthocyanin analysis, mobile phase A was 0.5% (*v*/*v*) water solution of phosphoric acid while mobile phase B was 100% HPLC grade methanol (Sigma Aldrich). Separation was optimized by gradient mobile condition as follows: linear from 3% to 65% B 0-38 min and 65% B 38–45 min with flow rate 1 mL min^−1^. The UV-Vis detector was set to monitor spectra from 190 to 600 nm while detection wavelength was 520 nm. For polyphenol analysis, mobile phase A was 0.1% (*v*/*v*) water solution of phosphoric acid while mobile phase B was 100% HPLC grade methanol (Sigma Aldrich). Gradient elution was performed as follows: linear 5–80% B from 0 to 30 min; 80% B from 30 to 33 min; linear 80–5% B, 33–35 min with flow rate 0.8 mL min^−1^. For both, anthocyanins and polyphenols determination operating conditions were: column temperature 20 °C, injection volume 10 µL of the standards and extract samples. The UV-Vis detector was set to monitor spectra from 190–600 nm while the detection wavelength was 360 nm. For identification the following standards were used: cyanidin 3-glucoside, cyanidin 3-arabinoside, cyanidin 3-galactoside, cyanidin 3-xyloside, myricetin, quercetin, chlorogenic acid and epicatechin (Sigma Aldrich). Individual anthocyanins and polyphenols were quantified using calibration curves and expressed as mg L^−1^.

### 3.8. Statistical Analysis

The obtained data were statistically analyzed in the software package SAS, version 9.3 [36]. Duncan’s test for significant difference (1%) was used. Results were subjected to one-way analysis of variance (ANOVA). The mean values were compared by *t*-test (LSD) and considered significantly different at *p* ≤ 0.0001. Correlation analysis was performed to investigate the nature and intensity of relation between two variables: the vitamin C content, total phenol, total flavonoid, total anthocyanin and antioxidant capacity. The correlation value was numerically expressed by Pearson correlation coefficient (*r*), while the coefficient significance was expressed by *p* value: * 0.01 < *p* < 0.05; ** *p* < 0.01; *** *p* < 0.0001, not significant at *p* > 0.05. 

## 4. Conclusions

Based on the results, can be concluded that addition of chokeberry powder increases the basic chemical composition parameters (density, total soluble solids, total acidity and pH) and bioactive compound levels (vitamin C, total phenols, total flavonoids, total anthocyanins) in juice samples regardless of the extraction method (conventional or high intensity ultrasound). Juice samples with added chokeberry powder also had higher values of antioxidant capacity regardless of the extraction method. High intensity ultrasound effects a significant increase of all studied nutritional parameters: vitamin C, total phenols, total flavonoids and total anthocyanins. The application of high intensity ultrasound significantly reduces the time required for extraction of the plant material given that after 30 min a significantly higher content of all analyzed bioactive compounds was achieved. A positive correlation between the content of bioactive compounds (vitamin C, total phenols, total flavonoids, total anthocyanins) and antioxidant capacity in juice samples treated with high intensity ultrasound was determined. Based on the stated evidence, must be emphasized that apple juice with added chokeberry powder, represents a highly nutritional valuable product with numerous potential benefits for human health due to its determined rich content of bioactive compounds and significant antioxidant capacity. Also, further research on this potential new product, especially studies oriented to determining the organoleptic characteristics of such a product are desirable to address various consumer and market demands.

## Figures and Tables

**Table 1:** Basic chemical composition of juice samples with added chokeberry powder extracted classically and by ultrasound.

Treatment	Density (g cm^−3^) *p* ≤ 0.0001	TSS (%) *p* ≤ 0.0001	TA (%) *p* ≤ 0.0001	pH NS
**Control Sample**				
A	1.0490 ^g^ ± 0.001	12.89 ^f^ ± 0.01	15.52 ^f^ ± 0.04	3.35 ± 0.01
**Classic Extraction**				
B1	1.0530 ^f^ ± 0.001	13.80 ^e^ ± 0.01	17.31 ^de^ ± 0.05	2.89 ± 0.70
B2	1.0527 ^f^ ± 0.001	13.73 ^e^ ± 0.01	17.10 ^e^ ± 0.07	3.37 ± 0.01
B3	1.0539 ^e^ ± 0.001	13.99 ^d^ ± 0.01	17.29 ^de^ ± 0.19	3.38 ± 0.01
B4	1.0539 ^e^ ± 0.001	14.02 ^cd^ ± 0.01	17.52 ^cd^ ± 0.24	3.38 ± 0.01
B5	1.0547 ^abc^ ± 0.001	14.22 ^a^ ± 0.01	17.70 ^abc^ ± 0.01	3.37 ± 0.01
B6	1.0543 ^cde^ ± 0.001	14.12 ^abcd^ ± 0.01	17.54 ^bcd^ ± 0.22	3.38 ± 0.01
B7	1.0540 ^de^ ± 0.001	14.06 ^bcd^ ± 0.01	17.65 ^abc^ ± 0.06	3.38 ± 0.01
**Ultrasonic Extraction**				
C1	1.0532 ^f^ ± 0.001	13.75 ^e^ ± 0.12	17.29 ^de^ ± 0.01	3.30 ± 0.03
C2	1.0541 ^de^ ± 0.001	14.01 ^d^ ± 0.08	17.66 ^abc^ ± 0.04	3.37 ± 0.01
C3	1.0545 ^abcd^ ± 0.001	14.09 ^abcd^ ± 0.08	17.71 ^abc^ ± 0.08	3.37 ± 0.01
C4	1.0543 ^bcde^ ± 0.001	14.06 ^bcd^ ± 0.02	17.59 ^abcd^ ± 0.14	3.37 ± 0.01
C5	1.0548 ^ab^ ± 0.001	14.16 ^abc^ ± 0.07	17.87 ^ab^ ± 0.04	3.35 ± 0.01
C6	1.0549 ^a^ ± 0.001	14.20 ^ab^ ± 0.04	17.93 ^a^ ± 0.05	3.37 ± 0.02

TSS—total soluble solids; TA—total acid content; NS—not significant. Different letters indicate significant differences between means.

**Table 2:** The content of bioactive compounds in juice samples with added chokeberry powder extracted classically and by ultrasound.

Treatment	Vitamin C (mg 100 g^−1^) *p* ≤ 0.0001	TPC (mg L^−1^) *p* ≤ 0.0001	TFC (mg L^−1^) *p* ≤ 0.0001	TAC (mg L^−1^) *p* ≤ 0.0001	Antioxidant Capacity (µmol TE L^−1^) *p* ≤ 0.0001
**Control Sample**					
A	13.16 ^i^ ± 0.88	512.64 ^k^ ± 0.09	205.89 ^m^ ± 0.40	ND	2164.54 ^e^ ± 0.01
**Classic Extraction**					
B1	12.83 ^i^ ± 0.86	828.04 ^i^ ± 0.70	328.99 ^l^ ± 0.91	687.88 ^h^ ± 3.04	2208.62 ^cd^ ± 0.03
B2	15.5 ^hi^ ± 1.68	829.23 ^i^ ± 0.51	360.47 ^i^ ± 0.59	714.02 ^gh^ ± 0.87	2213.79 ^bcd^ ± 0.01
B3	17.46 ^gh^ ± 0.85	831.16 ^hi^ ± 0.29	390.18 ^f^ ± 1.16	846.25 ^fg^ ± 0.87	2215.59 ^bcd^ ± 0.01
B4	20.57 ^fg^ ± 5.54	838.54 ^gh^ ± 0.19	350.62 ^k^ ± 0.43	919.12 ^ef^ ± 3.04	2228.86 ^abc^ ± 0.01
B5	22.49 ^ef^ ± 4.29	840.81 ^g^ ± 0.21	353.67 ^j^ ± 0.95	927.42 ^ef^ ± 1.73	2233.81 ^abc^ ± 0.01
B6	28.76 ^d^ ± 3.39	851.30 ^f^ ± 0.70	381.25 ^h^ ± 0.80	991.08 ^e^ ± 9.13	2237.36 ^abc^ ± 0.04
B7	36.78 ^bc^ ± 0.88	761.11 ^j^ ± 0.80	421.22 ^e^ ± 4.59	1579.94 ^bc^ ± 18.26	2189.05 ^de^ ± 0.01
**Ultrasonic Extraction**					
C1	15.57 ^hi^ ± 0.98	861.71 ^e^ ± 14.94	384.51 ^g^ ± 6.41	747.23 ^gh^ ± 15.65	2234.94 ^abc^ ± 0.01
C2	24.07 ^ef^ ± 3.92	894.04 ^d^ ± 14.64	443.01 ^d^ ± 1.15	997.54 ^e^ ± 56.53	2235.49 ^abc^ ± 0.01
C3	25.03 ^de^ ± 0.69	912.98 ^c^ ± 4.19	444.08 ^d^ ± 6.10	1393.90 ^d^ ± 0.44	2238.24 ^ab^ ± 0.01
C4	33.03 ^c^ ± 0.98	934.82 ^b^ ± 2.43	446.98 ^c^ ± 1.81	1452.02 ^cd^ ± 133.94	2239.55 ^ab^ ± 0.02
C5	40.87 ^ab^ ± 1.62	934.60 ^b^ ± 5.52	456.70 ^b^ ± 0.99	1707.86 ^ab^ ± 71.31	2241.84 ^a^ ± 0.01
C6	42.81 ^a^ ± 2.71	955.03 ^a^ ± 7.09	482.44 ^a^ ± 0.91	1823.79 ^a^ ± 77.85	2250.31 ^a^ ± 0.01

TPC—total phenol content; TFC—total flavonoid content; TAC—total anthocyanin content; ND—not determined. Different letters indicate significant differences between means.

**Table 3:** The anthocyanin profile and content of other polyphenols (mg L^−1^) determined in juice samples with added chokeberry powder.

Treatment	Cyanidin 3-Galactoside	Cyanidin 3-Glucoside	Cyanidin 3-Arabinoside	Cyanidin 3-Xyloside	Chlorogenic Acid	Epicatechin	Quercetin	Myricetin
	*p* ≤ 0.0001	*p* ≤ 0.0001	*p* ≤ 0.0001	*p* ≤ 0.0001	*p* ≤ 0.0001	*p* ≤ 0.0001	*p* ≤ 0.0001	*p* ≤ 0.0001
**Control Sample**								
A	ND	ND	ND	ND	6.23 ^k^	0.03 ^h^	0.04 ^l^	ND
**Classic Extraction**								
B1	34.34 ^m^	2.10 ^i^	4.37 ^l^	2.30 ^h^	6.47 ^j^	0.05 ^h^	0.08 ^k^	0.80 ^d^
B2	36.32 ^l^	2.10 ^i^	4.37 ^l^	2.30 ^h^	6.47 ^j^	1.22 ^g^	0.28 ^j^	0.80 ^d^
B3	44.84 ^j^	2.22 ^h^	5.54 ^j^	2.61 ^g^	7.71 ^i^	1.22 ^g^	0.58 ^h^	0.92 ^c^
B4	47.67 ^i^	2.34 ^g^	6.12 ^i^	2.61 ^g^	7.71 ^i^	1.22 ^g^	1.18 ^g^	0.92 ^c^
B5	49.09 ^h^	2.34 ^g^	6.26 ^h^	2.92 ^f^	8.34 ^h^	2.39 ^f^	1.28 ^f^	0.92 ^c^
B6	55.62 ^g^	2.58 ^f^	7.28 ^g^	3.22 ^e^	8.96 ^g^	2.99 ^e^	1.38 ^e^	1.04 ^b^
B7	93.93 ^c^	3.42 ^b^	12.67 ^c^	5.07 ^b^	13.33 ^d^	3.58 ^d^	2.18 ^c^	1.16 ^a^
**Ultrasonic Extraction**								
C1	42.56 ^k^	2.22 ^h^	5.09 ^k^	2.30 ^h^	6.47 ^j^	0.05 ^h^	0.08 ^k^	0.80 ^d^
C2	65.27 ^f^	2.70 ^e^	8.45 ^f^	3.53 ^d^	10.83 ^f^	2.99 ^e^	0.39 ^i^	0.80 ^d^
C3	85.98 ^e^	3.18 ^d^	11.51 ^e^	4.45 ^c^	12.08 ^e^	2.99 ^e^	1.78 ^d^	0.92 ^c^
C4	90.81 ^d^	3.30 ^c^	12.53 ^d^	5.07 ^b^	13.95 ^c^	4.16 ^c^	2.58 ^b^	0.92 ^c^
C5	110.39 ^b^	3.78 ^a^	15.44 ^b^	5.99 ^a^	15.82 ^b^	5.34 ^b^	2.58 ^b^	1.04 ^b^
C6	111.52 ^a^	3.78 ^a^	15.73 ^a^	5.99 ^a^	17.07 ^a^	5.93 ^a^	2.68 ^a^	1.04 ^b^

ND—not determined. Different letters indicate significant differences between means.

**Table 4:** Correlation coefficient (*r*) between the analyzed chemical compounds and antioxidant capacity (mmolTE L^−1^) in the apple juice samples with added chokeberry powder treated classical and by ultrasound.

Chemical Parameter	Correlation Coefficient (*r*)
**Classic Extraction**	
Vitamin C	−0.22 NS
Total phenols	0.90 ***
Total flavonoids	−0.44 NS
Total anthocyanins	−0.48 NS
**Ultrasonic Extraction**	
Vitamin C	0.83 *
Total phenols	0.82 *
Total flavonoids	0.79 *
Total anthocyanins	0.86 *

NS—not significant; ***—*p* ≤ 0.0001; *—0.01 ≤ *p* ≤ 0.05.

**Table 5:** Experimental design of classic and ultrasound extraction.

Extraction Method	Solvent	Solvent Volume (mL)	Time	Ultrasonic Bath	Sample
Classic	Apple juice	100	5 min	-	B1
Classic	Apple juice	100	10 min	-	B2
Classic	Apple juice	100	15 min	-	B3
Classic	Apple juice	100	20 min	-	B4
Classic	Apple juice	100	25 min	-	B5
Classic	Apple juice	100	30 min	-	B6
Classic	Apple juice	100	24 h	-	B7
UAE	Apple juice	100	5 min	35 kHz 140 W	C1
UAE	Apple juice	100	10 min	35 kHz 140 W	C2
UAE	Apple juice	100	15 min	35 kHz 140 W	C3
UAE	Apple juice	100	20 min	35 kHz 140 W	C4
UAE	Apple juice	100	25 min	35 kHz 140 W	C5
UAE	Apple juice	100	30 min	35 kHz 140 W	C6
